# Physicians’ Hand-Book of Practice for 1864

**Published:** 1864-01

**Authors:** William Elmer, W. A. Townsend

**Affiliations:** New York; Publisher, 39 Walker Street


*Physicians' Hand-Book of Practice for* 1864, *by* WILLIAM ELMER, M. D. *New York:* W. A. TOWNSEND, *Publisher*, 39 *Walker Street.*

The following is the Table of Contents: Preface—Classification of Diseases—Fevers—Eruptive Fevers—Constitutional Diseases—Diseases of Blood and Blood-Vessels—of Brain, Spinal Cord and Nerves—Diseases of Respiratory Organs—of Circulating Organs—Digestive and Absorbent Systems—of Internal Organs of Secretion—Cutaneous Diseases—Diseases of the Re-productive Organs—Midwifery and the Diseases of Women—Ready Method in Asphyxia—Poisons and their Antidotes—Diagnostic Examination of the Urine—The Pulse, Table of, &c.—List of Incompatibles—Medicinal Weights and Measures—Abreviations of Medicinal Properties of Remedies—Materia Medica—Index of Common Names of Remedical Agents—Extemporaneous Prescriptions: Remarks and Examples—Explanation of Signs, &c., to be used in the Record—Names and Addresses—Bills and Accounts—Record of Practice and Treatment—Obstetric Calendar—Obstetric Record—Wants and general Memoranda—List of Nurses.

This is the most complete "Hand-Book of Practice," published. In all its arrangements it is unexceptionable. It is remarkable how much is crowded into a Pocket Memorandum Book, which is almost indispensable to the physician. They are for sale in Buffalo by BREED, BUTLER & Co.

